# Comparison of Functional Outcomes and Sagittal Balance in Adolescent Idiopathic Scoliosis Corrective Surgery Utilizing Conventional Rods and Patient-Specific Rods: Preliminary Results

**DOI:** 10.7759/cureus.105548

**Published:** 2026-03-20

**Authors:** Foo Kuok Thong, Khoh Phaik Shan, Han Sim Lim, Zairul Bahrin, Marazuki Bin Haji Perwira, Rajandra Kumar Karupiah

**Affiliations:** 1 Orthopedics, Hospital Pulau Pinang, Georgetown, MYS; 2 Orthopedics, Hospital Sultanah Bahiyah, Alor Setar, MYS; 3 Spine Surgery, Hospital Tengku Ampuan Afzan, Kuantan, MYS; 4 Orthopedics, Traumatology, and Rehabilitation, Kulliyyah of Medicine, International Islamic University Malaysia, Kuantan, MYS

**Keywords:** adolescent idiopathic scoliosis, conventional rods, functional outcome, patient-specific rods, sagittal balance

## Abstract

Background: Adolescent idiopathic scoliosis (AIS) is a three-dimensional spinal deformity that arises in otherwise healthy children. Surgical correction for patients with a Cobb angle is usually performed with posterior corrective surgery utilizing rods bent intraoperatively. With the invention of patient-specific rods (PSR), we compared the radiological and functional outcomes of patients operated on with either rod.

Method: A retrospective cohort study in which AIS patients who underwent posterior corrective surgery from April 2019 until March 2021 in two state hospitals were recruited. They were divided into two groups based on the rods utilized: PSR and conventional rod (CR). Radiographic evaluation of sagittal vertical axis (SVA), pelvic tilt (PT), and pelvic incidence-lumbar lordosis mismatch (PI-LL) parameters was recorded before and three months after the operation. Surgeries were performed with the aim of achieving SVA <50 mm, PT<20°, and PI-LL<10°. Patients were interviewed using SRS-30 questionnaires six months postoperatively to compare the functional outcome.

Results: Thirty-three patients were recruited; 18 were in the PSR group, and 15 were in the CR group. Pre- and postoperative PI-LL change in the CR group is significant (p<0.05). Comparison of mean difference showed that only the PI-LL difference is significant between PSR and CR (p<0.05). The PSR group showed a higher percentage of achieving planned correction in all three parameters, with 13 patients (72.22%) in PI-LL, 94.44% in PT, and 17 patients (94.44%) in SVA. In the CR group, only SVA had a higher percentage of patients able to achieve planned sagittal parameters. Both groups produced comparable functional outcome scores, with a total average of 4.36 for PSR and 4.47 for CR, out of a maximum of 5.

Conclusion: PSR can achieve better planned sagittal parameters compared with CR clinically. Both PSR and CR groups showed comparable functional outcomes.

## Introduction

Adolescent idiopathic scoliosis (AIS) is a complex three-dimensional deformity characterized by a minimum lateral spine deviation of 10°, associated with rotation of the vertebrae, with or without hypokyphosis [1]. AIS is a diagnosis of exclusion, which means other causes of scoliosis, such as syndromic disorders, vertebral malformations, and neuromuscular disorders, have been ruled out. The term "idiopathic" indicates that the disorder has no identified cause, but major studies, including those on the genetic basis of AIS, are ongoing. It affects around 1-3% of children in the at-risk population aged 10-16 years [2].

Sagittal spinal alignment is important in making the human erect position energetically economical and sustainable. In AIS, sagittal spine alignment is often different compared with that of normal subjects due to structural deformity of the spine. Compensation mechanisms for sagittal spinal malalignment, such as knee flexion and a retroverted pelvis, lead to difficulties in maintaining an upright standing position, reduced walking distance, and forward bending. Thus, analysis of sagittal alignment is crucial in corrective scoliosis surgery. Three clinically relevant parameters were proposed by Schwab et al. to simplify the analysis and set targets for realignment surgery. The parameters are as follows: difference between pelvic incidence (PI) and lumbar lordosis (LL) <10°, sagittal vertical axis (SVA) <50 mm, and pelvic tilt (PT) <20° [3]. SVA restoration makes it easier to maintain a level gaze and adopt a physiological standing position. The C7 plumb line moves behind the femoral heads with an SVA of <50 mm, which alleviates the complaint of "falling forward." To reestablish proper femoral pelvic-spinal alignment, which is necessary for effective ambulation, PT realignment to <20° is highly recommended. This parameter should be properly realigned because it has been separately demonstrated to correspond with deterioration in walking tolerance. By achieving a mismatch of PI-LL <10°, the angulation of the hypolordotic spine increases to match the patient’s spinopelvic morphotype (i.e., PI), which restores the appropriate lordotic alignment. Patient outcomes improve when these parameters are achieved through surgery [4].

AIS patients are reported to have a variety of health issues, such as lower quality of life, back discomfort, pulmonary dysfunction, stress, and mental health issues. The difficulty for researchers is choosing a tool that is sensitive, specific, and reliable while still capturing the distinctive features of the disease and the outcome after intervention. Physical functioning, or functional outcome, is a key factor in health status and health-related quality of life, and it can be used to identify people who are at risk of impairment and forecast who will need health and social care. It can be assessed via several methods, such as patient-reported outcome measures or performance-based outcome measures. The Scoliosis Research Society (SRS) questionnaire and its variations are the most often used patient-reported outcome measures for evaluating the quality of life and physical functioning of people with spinal deformities [5]. The SRS-30 questionnaire was utilized as it was the latest edition of the questionnaire during the period when the study was carried out.

Even with established tools for surgical planning, sagittal spinal alignment is not always adequately achieved postoperatively. Most rods are contoured by surgeons using their own experience, without measuring the rod’s angle or determining the desired amount of kyphosis. Although multiple steps influence final alignment, we postulate that manual bending of a rod cannot always provide the intended rod contour because it introduces an operator-dependent component. Thus, with the introduction of patient-specific rods (PSR), it is postulated that the inaccuracy due to unmeasured intraoperative rod bending will be reduced. Studies have shown that rods that are prebent industrially according to patient-specific data are effective in correcting spinal deformities in adults.

## Materials and methods

**Figure 1:**
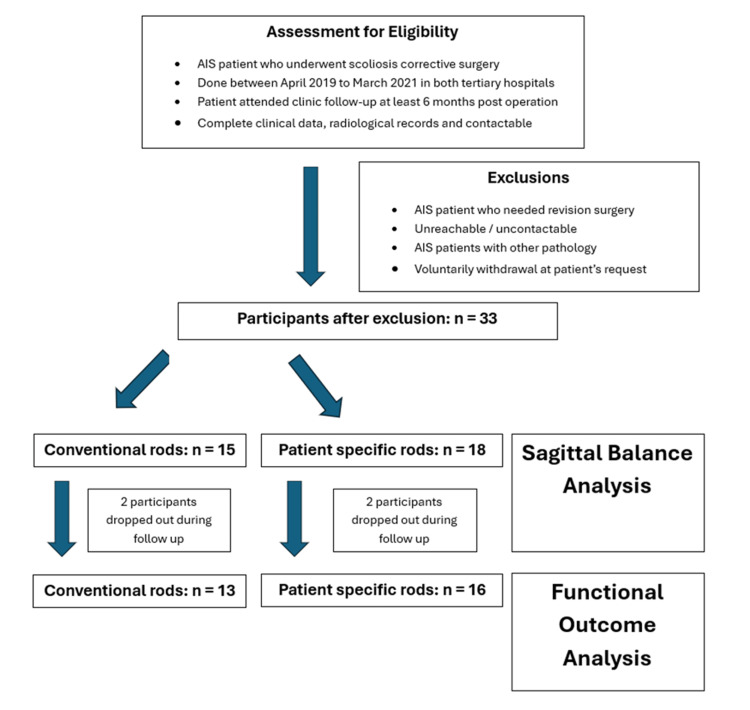
CONSORT flow diagram depicting the recruitment process and the number of patients in each group CONSORT, Consolidated Standards of Reporting Trials; AIS, adolescent idiopathic scoliosis

This retrospective cohort study included all AIS patients, including both genders and all ethnic groups, who underwent posterior corrective scoliosis surgery in two tertiary state hospitals, operated on by a single spine surgeon between April 2019 and March 2021. The study aimed to compare sagittal correction radiologically and functional outcomes in patients utilizing either one of the rods. Patients with triple major curve type scoliosis or underlying pathology, such as hyperlaxity, dextrocardia, and meningomyelocele, were excluded from the study (Figure 1). Spinopelvic parameters were measured from calibrated whole-spine standing lateral-view X-ray images in patients who satisfied both inclusion and exclusion criteria. For PSR, preoperative sagittal parameters were used, and surgical procedures were simulated using UNiD Hub version 4.2.4 (UniD™, USA), aimed to achieve the following parameters: SVA <50 mm, PT <20°, and PI-LL <10°. A pair of PSRs would be manufactured based on these specifications industrially by MEDICREA®, a company based in Lyon, France, and the rods were contoured based on the virtually corrected spine. As for CR, the rods were manually contoured using whole-spine X-rays as a guide with the aim of achieving the same objectives. The surgical technique used was the rod derotation method with facetectomy, with or without additional maneuvers such as direct vertebral rotation and compression-distraction force whenever deemed necessary intraoperatively [6]. Patients were scheduled for follow-up appointments at postoperative one, three, and six months. Radiographic images were measured for sagittal parameters using the measurement tool of the workstation in RadiAnt™ DICOM Viewer (Medixant, Poland) by investigators for all parameters and documented in degrees of angle at six months postoperation. If the postoperative parameters fell within the acceptable range, then the objectives were considered achieved, regardless of the preoperative parameters.

The patients’ functional outcomes were also evaluated in this study. This study utilized the free-to-use SRS-30 questionnaire by SRS, which comprised five domains, including function/activity (seven questions, maximum 35 points), pain (six questions, maximum 30 points), self-image/cosmesis (nine questions, maximum 45 points), mental health (five questions, maximum 25 points), and satisfaction with management (three questions, maximum 15 points). The total scores were divided by the number of questions answered in each domain, giving the highest mean score of 5. Higher scores indicate better outcomes in each domain individually. Patients’ functional outcomes were assessed after six months postoperatively because studies showed that more than half of patients have achieved adequate bone and wound healing and likely will be able to return to their normal physical activity. Interviews were also conducted using the questionnaire in the same setting or via teleconference if the patient could not attend clinic follow-ups. The results were collected, tabulated, and analyzed using the SPSS® Statistics tool version 25 (IBM, New York, USA). The categorical data were analyzed using the Chi-square/Fisher exact test, while the Wilcoxon signed rank test was used to analyze the nonparametric data. Spearman’s rank correlation coefficient was used to analyze the correlation between radiographic parameters of both types of rods and all domains in the functional outcome assessment.

The study was conducted after attaining ethical approval from the Medical Research & Ethics Committee (MREC) of the Ministry of Health (MOH), Malaysia, via the National Medical Research Register (NMRR-20-3169-56116). Ethical approval was also obtained from the Research and Ethics Committee (IREC: IIUM/305/20/4/1/7). Written consent and permission were also obtained from the hospital directors before the study was conducted. The study was also conducted in compliance with the ethical principles outlined in the Declaration of Helsinki and the Malaysian Good Clinical Practice Guideline.

## Results

A total of 33 patients were recruited for this. There were 18 patients in the PSR group and 15 patients in the CR group. Out of this, two from each group dropped out because either the patient did not turn up for subsequent follow-up or was uncontactable. The patients’ mean age was 16.12±3.66, with the youngest being 10 years old and the oldest being 31 years old. The mean weight for the patients was 45.97±10.96 kg, and the mean height was 151.45±12.22 cm. The average Cobb angle was 72.79±18.74°. The summary of the patients’ demographic data is shown in Table 1.

**Table 1:** Demographic data of patients participating in this study (n=33) ^a^Independent t-test PSR, patient-specific rod; CR, conventional rod

Demographic data	Frequency (%)			
	CR		PSR	
Male	0 (0)		4 (12.12)	
Female	15 (45.45)		14 (42.42)	
Malay	13 (39.39)		14 (42.42)	
Chinese	2 (6.06)		3 (9.09)	
Indian	0 (0)		1 (3.03)	
	Mean (standard deviation)			p-value^a^
Age (years old)	16.31 (2.30)	15.94 (4.67)		0.776
Weight (kg)	43.53 (7.30)	48.21 (13.41)		0.227
Height (cm)	152.63 (6.97)	150.29 (15.80)		0.592
Cobb angle (°)	73.06 (19.46)	72.53 (18.62)		0.936

Among the total of 33 patients, 18 of them underwent posterior corrective scoliosis surgery utilizing PSR (Table 2). The PI-LL mismatch had a median of 5° for both preoperative (14.75) and postoperative (7.50) assessments. The median for PT was 11.50° (9.05) and 5 mm (38.75) for SVA, respectively. When preoperative and postoperative values were compared for all three sagittal parameters, the change was statistically insignificant (p>0.05). As for the remaining 15 patients who were operated on using CR, the preoperative PI-LL median was 0° (15.00) and 9.00° (9.00) postoperatively. The median for PT and SVA preoperatively were 9.00° (10.00) and -5 mm (35.00). After the surgery, we obtained a median of 15.00° (8.00) for PT and 0 mm (21.00) for SVA. However, after analyzing the results for both preoperative and postoperative values, the change in PI-LL was statistically significant (p<0.05), while the remaining two parameters were statistically not significant (p>0.05).

**Table 2:** Comparison of preoperative and postoperative sagittal parameters in PSR and CR ^a^Mann-Whitney test ^*^Result is significant (p<0.05) PSR, patient-specific rod; CR, conventional rod; SVA, sagittal vertical axis; PT, pelvic tilt; PI, pelvic incidence; LL, lumbar lordosis

Type of rods	Outcome measures	N	Preoperative assessment (median/IQR)	Postoperative assessment (median±IQR)	p-value^a^
PSR	PI-LL (degrees)	18	5.00/14.75	5.00/7.50	0.793
	PT (degrees)	18	11.50/9.05	13.00/6.50	0.622
	SVA (mm)	18	5.00/38.75	17.00/35.75	0.589
CR	PI-LL (degrees)	15	0.00/15.00	9.00/9.00	0.009*
	PT (degrees)	15	9.00/10.00	15.00/8.00	0.069
	SVA (mm)	15	-5.00/35.00	0.00/21.00	0.649

Table 3 shows the comparison of the mean difference in preoperative and postoperative measurements in both groups. In the PSR group, the mean difference for PI-LL was 5.67° (6.37), 6.17° (6.25) for PT, and 20.17 mm (19.69) for SVA, whereas in the CR group, the mean difference for PI-LL was 10.60° (±6.69), 8.33° (±5.85) for PT, and 29 mm (±23.53) for SVA. Statistical analysis with the Mann-Whitney test showed the mean difference of PI-LL between the two rods was statistically significant (p=0.01), while the mean differences of PT and SVA when comparing both rods were not statistically significant (p>0.05).

**Table 3:** Comparison of the mean difference in preoperative and postoperative measurements between the PSR and CR groups ^a^Mann-Whitney test *Result is significant (p<0.05) PSR, patient-specific rod; CR, conventional rod; SVA, sagittal vertical axis; PT, pelvic tilt; PI, pelvic incidence; LL, lumbar lordosis

Outcome measures	PSR		CR		p-value^a^
	N	Mean±SD	N	Mean±SD	
PI-LL (degrees)	18	5.67±6.371	15	10.60±6.685	0.013*
PT (degrees)	18	6.17±6.252	15	8.33±5.851	0.210
SVA (mm)	18	20.17±19.690	15	29.00±23.528	0.238

In addition, we also recorded the number and percentage of patients who achieved the desired sagittal balance parameters, as tabulated in Table 4. Patients operated on with PSR had a higher percentage in achieving the objective, as 13 patients (72.22%) achieved the desired results for PI-LL, compared with eight patients (53.33%) in the CR group for the same parameter. The other two sagittal parameters also showed a higher percentage in the PSR group compared with the CR group. In the PSR group, 17 patients (94.44%) were able to achieve the targeted PT value, with only one patient (5.56%) unable to achieve the desired PT value postoperatively. A similar result was seen in the PSR group for the SVA parameter, where only one patient’s (5.56%) SVA value was not within the desired range. However, the result in the CR group for PT was less favorable, as only 10 patients (66.67%) were able to achieve the targeted range. As for SVA in the CR group, we were able to correct 14 out of 15 patients (93.33%) to <50 mm postoperatively. When the results were analyzed statistically with the Pearson chi-square test and Fisher’s exact test, all three parameters were statistically insignificant.

**Table 4:** Comparison of surgical objectives achieved between the PSR and CR groups ^f^Fisher's exact test (when more than 20% have an expected count of less than 5) ^c^Pearson chi-square test PSR, patient-specific rod; CR, conventional rod; SVA, sagittal vertical axis; PT, pelvic tilt; PI, pelvic incidence; LL, lumbar lordosis

Outcome measures	PSR (n=18), n (%)	CR (n=15), n (%)	p-value
Objective achieved - PI-PL	13 (72.22)	8 (53.33)	0.261^c^
Objective achieved - PT	17 (94.44)	10 (66.67)	0.070^f^
Objective achieved - SVA	17 (94.44)	14 (93.33)	1.000^f^

The results of postoperative functional outcomes of the patients assessed using the SRS-30 questionnaire were summarized in Table 5. The median total SRS score was 4.36 (0.50) for the PSR group and 4.47 (0.22) for the CR group postoperatively. The results were analyzed with the Mann-Whitney test, and we found that functional outcome scores between the two rods in all domains were statistically insignificant.

**Table 5:** Postoperative functional outcome assessment in the PSR and CR groups ^a^Mann-Whitney test PSR, patient-specific rod; CR, conventional rod

Functional outcomes	PSR		CR		p-value^a^
	N	Median/IQR	N	Median/IQR	
Function	16	4.00/0.65	13	4.14/0.43	0.453
Pain	16	4.33/0.96	13	4.33/0.50	0.350
Self-image	16	4.17/0.67	13	4.39 /0.56	0.179
Mental health	16	4.76/0.40	13	4.67/0.40	0.946
Satisfaction	16	4.33/0.67	13	4.67/0.67	0.254
Total average score	16	4.36/0.50	13	4.47/0.22	0.219

Using Spearman's rank correlation, we analyzed the correlation between the five domains in the functional outcome assessment. There was a positive and moderate correlation between function and pain score (r=0.535, p=0.003). Function also had a positive but weak correlation with the mental health domain (r=0.435, p=0.018). The satisfaction score had a positive and good correlation with self-image (r=0.772, p<0.001) but a weak positive correlation with the pain domain (r=0.401, p=0.031). The results were summarized in Table 6.

**Table 6:** Correlation between functional outcome scores r, correlation coefficient

Functional outcomes		Function	Pain	Self-image	Mental health	Satisfaction
Function	r (p-value)	1	0.535 (0.003)	0.240 (0.210)	0.435 (0.018)	0.108 (0.575)
Pain	r (p-value)	0.535 (0.003)	1	0.345 (0.066)	0.281 (0.140)	0.401 (0.031)
Self-image	r (p-value)	0.240 (0.210)	0.345 (0.066)	1	0.303 (0.111)	0.772 (<0.002)
Mental health	r (p-value)	0.435 (0.018)	0.281 (0.140)	0.303 (0.110)	1	0.314 (0.097)
Satisfaction	r (p-value)	0.108 (0.575)	0.401 (0.031)	0.772 (<0.001)	0.314 (0.097)	1

As a summary of the study results, the use of PSR produced a higher percentage of patients who successfully achieved the desired sagittal parameters compared with CR clinically. However, functional outcome assessment of patients in both groups did not show any statistically significant difference.

## Discussion

In this retrospective cohort study, our results showed that PSR was able to produce radiological outcomes comparable to conventionally bent rods (CR). Analysis of mean differences in preoperative and postoperative measurements between both groups also yielded similar results, with the PI-LL parameter (p<0.05) being the only statistically significant difference between PSR and CR. However, when considering the percentage of patients who achieved the planned sagittal parameters, the PSR group produced better results compared with the CR group. This finding corresponds with a recent study by Jabbouri SS [7]. Although that study evaluated additional sagittal parameters beyond PI-LL mismatch, including thoracic kyphosis and T1 pelvic angle, the mean difference between AI-predicted and observed values in the PSR group was close to the target value. The authors also concluded that PSR technology is able to execute preoperative surgical planning more accurately in postoperative outcomes, as evidenced by the higher percentage of PSR patients achieving desired parameters. Solla F et al. demonstrated that PSR can be used to restore thoracic kyphosis in hypokyphotic patients with better accuracy, while Prost et al., in another paper, mentioned that PSR may help reduce the proportion of surgically undercorrected patients due to insufficient planning or poor rod bending [8,9].

Several studies, including ours, revealed that PSR has multiple advantages over CR. PSR rods are industrially bent and notch-free. These rods are better at maintaining their curvature and thus spinal correction compared with notched rods, which typically occur during manual bending with a French bender. Prior research revealed that notch-free rods had a substantially greater ultimate load than notched rods while maintaining the same stiffness, suggesting that notch-free rods are more likely to retain their form [10]. This can be explained by the mechanical effects of notches, which create local stress exceeding the material’s yield strength and leading to plastic deformation. Since rod breakages mostly occur at stress concentration points, notches play a significant role in fatigue failure. In most cases, rods bent with a French bender are recontoured repeatedly intraoperatively, further decreasing their fatigue strength. Thus, notch-free rods should be more durable and less prone to breakage compared with conventional rods (CRs), as reported by Sudo H et al. and Fiere V et al. [11,12].

The application of PSR is supported by computer software such as UniD™ (Minneapolis, USA), which assists in operative planning of surgical realignment. These programs can simulate spinal correction with planned rod curvatures and various osteotomies [11]. This greatly reduces operative time while improving the accuracy and consistency of spinal correction.

This study showed no significant differences in functional outcome assessments between the PSR and CR groups. Patients operated on with CR had slightly higher average total scores compared with those with PSR, but this difference was neither clinically nor statistically significant (p>0.05). This may be attributed to the relatively short follow-up duration, as assessments were conducted only up to six months postoperatively. By contrast, Solla F et al.’s analysis of 27 AIS patients corrected with PSR showed improvement in SRS scores from 3.64 to 4.27 after surgery [13].

Although this study suggests that functional outcomes do not directly correspond to radiological parameters, a finding supported by Hisam et al., who also reported no direct correlation between patient satisfaction and radiological outcomes, other studies have shown mixed results [14]. Lafage V et al. suggested that Health-Related Quality of Life (HRQOL) measurements and sagittal plane characteristics show significant correlation [4]. Their study also reported that PT correlated with increased pain, one of the domains in the SRS questionnaire for adult spinal deformity. However, most studies supporting such correlations were conducted in adult populations with spinal deformity. Since most AIS surgeries aim to achieve optimal sagittal parameters and global sagittal balance, often guided by the SRS-Schwab and Roussouly classifications, Ilharreborde argued that the goals outlined by Schwab et al. in ASD cannot be directly translated to AIS [15]. A recent narrative review by Bautista AG et al., compiling several studies utilizing PSR in both ASD and AIS, summarized that PSR demonstrated promising preliminary clinical outcomes in AIS patients, with up to a 74% reduction in coronal Cobb angle [16].

The limitations of this study include variation and measurement accuracy of radiological parameters, since examinations were performed by different personnel and were subject to radiographic projection effects. Although the follow-up period used in this study was supported by the literature, it was still relatively short compared with other studies. Future research with larger, multicenter, randomized samples and longer follow-up periods may yield results that better evaluate the advantages and disadvantages of PSR compared with CR.

## Conclusions

PSR was able to produce better results in achieving planned sagittal parameters compared with CR. Regarding functional outcomes, the PSR group showed comparable results to the CR group. Although PSR did not demonstrate significant benefits or improvements over CR in this study, the technology is safe and reliable for use in treating AIS patients, offering an alternative to conventional CR. Throughout the study period, no implant-related complications were observed. We conclude that the use of PSR in posterior scoliosis corrective surgery is noninferior to CR, as highlighted by recent literature. However, further studies are needed to strengthen the evidence supporting the potential advantages of PSR technology in the future.
